# Supplementary material for: INHIBITION OF THE PROSTAGLANDIN-DEGRADING ENZYME 15-PGDH AMELIORATES MASH-ASSOCIATED APOPTOSIS AND FIBROSIS IN MICE
Source: Cells. 2025 Jun 27;14(13):987. doi: 10.3390/cells14130987 (PMC12248568; doi:10.3390/cells14130987)
Supplement: Supplementary file 1 [file cells-14-00987-s001.zip › cells-3558071-Supplemental Material.pdf]

SUPPLEMENTAL MATERIAL

EXPANDED METHODS

Plasma Treatment. Heparinized blood, collected in glass tubes, was cooled by gentle repeated inversions in an ice water slurry for 1min and centrifuged (3,000rpm x 10min at 4°C). To prevent its oxidation, GSH was immediately converted to a stable thioether by treating 100µl of blood with 100µl of 50mM iodoacetate in 10mM ammonium bicarbonate, pH=10, adjusted with concentrated ammonia hydroxide. After the buffy layer and the red blood cell pellet were removed, aliquots of plasma were treated with 50µl of iodoacetate buffer (vol:vol, 1:1), collected in pre-labeled microtubes and quick-frozen to be stored at -80°C until analysis.

Liquid Chromatography-Mass Spectrometry (LC-MS) for Glutathione sp. Reduced and oxidized glutathione (GSH and GSSG) in plasma were analyzed using methods validated previously with minor modifications. [1-4] In brief, plasma samples were first treated with iodoacetate to derivatize GSH as GS-carboxymethyl, then GSSG in plasma were converted to GS-cyanomethyl using iodoacetonitrile after dithiothreitol reduction. The internal standard homo- glutathione was spiked at the beginning of the process. All calibration curves consisted of two blanks and seven calibration points. The curve ranges were as follows: GSH, 0.78 – 200µM; GSSG, 0.157 – 40µM. A weighting factor of 1/x2 was applied over the calibration curves.

The resulting peak area ratios of analyte/internal standard were plotted against the concentrations. Electrospray-ionization mass spectrometry of thioethers was performed on a Thermo Scientific TSQ Quantum Ultra mass spectrometer (Thermo Fisher Scientific, Waltham, MA), equipped with a heated electrospray ion source (HESI-II), coupled to an Agilent 1200 HPLC. The chromatography was done with a reversed-phase C18 column (Synergi 4µ Hydro-RP, 50x2.0mm, Phenomenex). The compounds of interest were separated from mouse plasma endogenous components using 1% acetonitrile containing 0.1% formic acid at 0.1ml/min isocratic. The mass spectrometry was operated under positive ionization mode with the ion spray voltage at 4000V. The ion transfer tube temperature was maintained at 400°C, and the vaporizer temperature was 40°C. The gas setting for sheath, aux, and ion sweep were 50, 2, and 0 arb, respectively. Argon gas pressure was 1.5mTorr used as the collision gas in Q2. Thecollision energy was 15V for monitored transitions. Peak width for Q1 and Q3 was set at 0.7FWHM. Xcalibur software (version2.1.0, Thermo) was used for data registration. The area under the curve of the spectra was recorded and captured on a database for concentration calculations and data analyses. Similar procedure was used for assessment of glucose concentration.

Liquid Chromatography-Mass Spectrometry (LC-MS) for Non-Targeted Metabolon. Solvent extraction from thaw samples was performed with MAA (methanol: acetonitrile: acetone; 1:1:1) and internal standards were reconstituted with methanol:H_2O/2:98/vol:vol. LC-MS analyses were performed on a 1290 Infinity Binary LC system from Agilent used for chromatographic separation in conjunction with a Waters Acquity UPLC HSS T3 1.8µm 2.1x100mm column in connection with a Water Acquity UPLC HSS T# 1.8µm pre-column. The column temperature

was set up at 55°C at a flow rate of 0.45ml/min with time intervals for system equilibration (7min) and data acquisition (27min) for a total run time of 34min. Mobile phase-A was 0.1% formic acid in the water, and mobile phase-B was 0.1% formic acid in methanol. For elution, mobile phase-A and B were initially held at 98%:2% for 20min. Then, from 20.1min, the mobile phase was brought to 25%-A: 75%-B and held there for 2 min. Then, from 22.1min, the eluent was brought to 2%-A and held there until 30min. Finally, from 30.1 to 37min, the eluent was bought back for re-equilibration to 98%-A. Positive and Negative mass spectra curves were acquired in scan mode with a mass range of 50 to 1000m/z. Inline calibration was performed using debrisoquine sulfate (m/z 176.1182) and HP-0921 from Agilent (m/z 922.0098) in the positive mode, and 4-NBA (m/z 166.0146) and HP-0921 from Agilent (m/z 966.0007 formate adduct) in the negative mode. Mass spectrometer was set up as follows: gas temperature of the ion source at 325°C with drying gas flow at 10 l/ml; the nebulizer pressure was 45psi with a sheath gas at 400°C, a sheath flow of 12 l/ml and capillary voltage of 4000V, fragmentor voltage at 140V, and skimmer voltage at 65V. Raw data were deconvoluted with the National Institute of Standards and Technology (NIST) Automated Mass Spectral Deconvolution and Identification Software (AMDIS). After spectral analysis and data processing of ≈ 800 signals, 94 signals could be identified in 89% of all samples. Identified signals were confirmed by our metabolomic library and the Fiehn library (Agilent Technologies Inc, Santa Clara, CA). For further quantification, the data was exported to the University of Michigan Core Metabolomic Server. The concentration of each metabolite was expressed as its relative peak area (divided by the area of the corresponding internal standard in the same chromatogram). Some small compounds, although they were specifically targeted, were not found or identified with certainty in the present model. They included glycerol, pyruvate, and aceto-acetone. All 94 identified metabolites were included in the statistical analyses.

Hematoxylin and Eosin (H& E) Staining. For H&E, after deparaffinization and rehydration procedure, slides were immersed in filtered Harris hematoxylin for 2 min, rinsed with water and then immersed in 0.3% ammonium hydroxide (10-20 dips). Slides were then rinsed with water and immersed in Eosin-Y (10 dips). Slides were washed with water and dehydrated in the following order of ethanol 70% EtOH for 1 min, 80% EtOH for 1 min, 95% EtOH for 1 min (thrice) and then100% EtOH for 1 min (thrice). Following which the slides were immersed in xylene for 1 min (twice) and coverslipped with Permount.

Trichrome staining. For Trichrome staining, after deparaffinization and rehydration procedure, slides were placed in 40mL of Bouin's fluid in a plastic coplin jar with lid applied loosely, microwave on high for 30 seconds and allow to stand for 5 minutes. Thereafter the slides were rinsed in tap water for 5 minutes until yellow color was cleared. They were then placed in Working Weigert's Iron Hematoxylin Stain for 10 minutes and rinsed in tap water for 10 minutes. After which they were placed in trichrome stain for 15 minutes and moved into 1% Acetic Acid Solution for 1 minute. Slides were then rinsed in deionized water for 30 minutes, dehydrated

through two changes of 100% ethanol (1 minute each) and cleared in three changes of clearing agent (1min each). Cover slipped with Permount and stored overnight at room temperature to allow mounting medium to set up before imaging.

References

1. Andres Ibarra R, Abbas R, Kombu RS, Zhang GF, Jacobs G, Lee Z, et al. Disturbances in the glutathione/ophthalmate redox buffer system in the woodchuck model of hepatitis virus- induced hepatocellular carcinoma. HPB Surg. 2011; 2011:789323.
2. Ibarra R, Dazard JE, Sandlers Y, Rehman F, Abbas R, Kombu R, et al. Metabolomic Analysisof Liver Tissue from the VX2 Rabbit Model of Secondary Liver Tumors. HPB Surg. 2014; 2014:310372.
3. Kombu RS, Zhang GF, Abbas R, Mieyal JJ, Anderson VE, Kelleher JK, et al. Dynamics of glutathione and ophthalmate traced with 2H-enriched body water in rats and humans. Am J Physiol Endocrinol Metab. 2009;297(1): E260-9.
4. Sanabria JR, Kombu RS, Zhang GF, Sandlers Y, Ai J, Ibarra RA, et al. Glutathione species and metabolomic prints in subjects with liver disease as biological markers for the detection of hepatocellular carcinoma. HPB (Oxford). 2016;18(12):979-90.

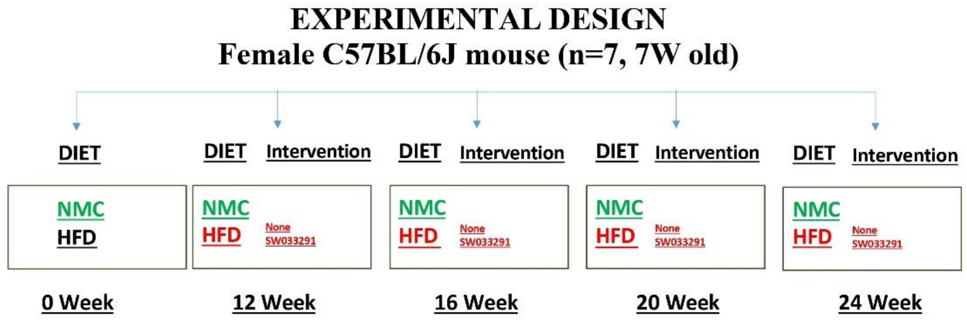

Figure S1. *Experimental design flowchart for the MASH mouse model.* The chart displays the timeline and interventions for the MASH mouse model experiments where mice were fed ad libitum with normal mouse chow (NMC) or a high fat diet complemented with 55% fructose-in-water (HFD). After 12 weeks, the rodents were divided into NMC, HFD, and HFD±SW033291 groups. The control, HFD and HFD± SW033291 groups were maintained for another 4 weeks (study time point 16W), 8 weeks (study time point 20W) and 12 weeks (study time point 24W), when the study was concluded.

Figure S2a.

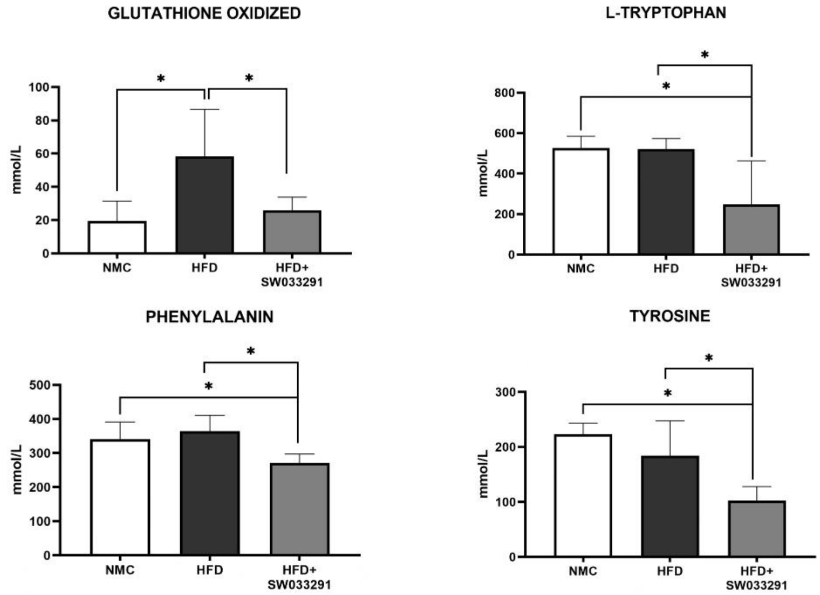
GLUTATHIONE OXIDIZED
L-TRYPTOPHAN
PHENYLALANIN
TYROSINE
mmol/L
NMC
HFD
HFD+
SW033291
*

Figure S2b.

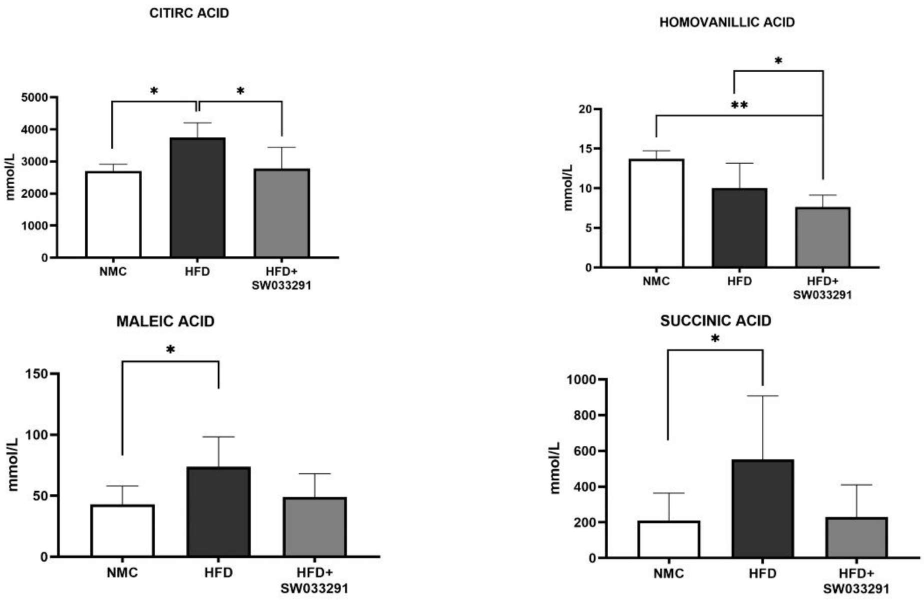

Figure S2c.

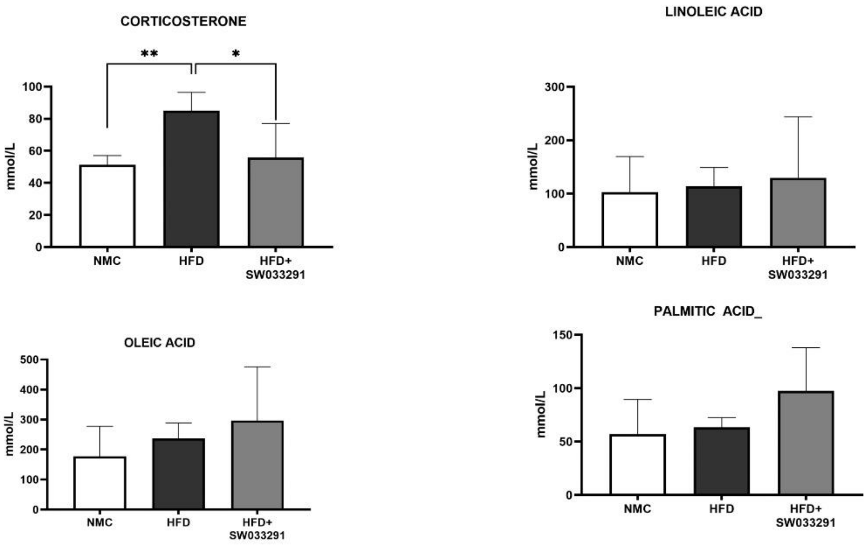

Figure S2 (a-c). *Metabolomics data.* Plasma concentration of non-targeted metabolites for each experimental group at 24W assayed with LC/MS-MS technique. Observe the significant increase in the glutathione oxidized, citric acid and corticosterone in the HFD mice compared both the NMC group and SW033291-treated mice. (* $p < 0.05$, ** $p < 0.01$, by ANOVA and t-test, $n = 5$)

Figure S3a.

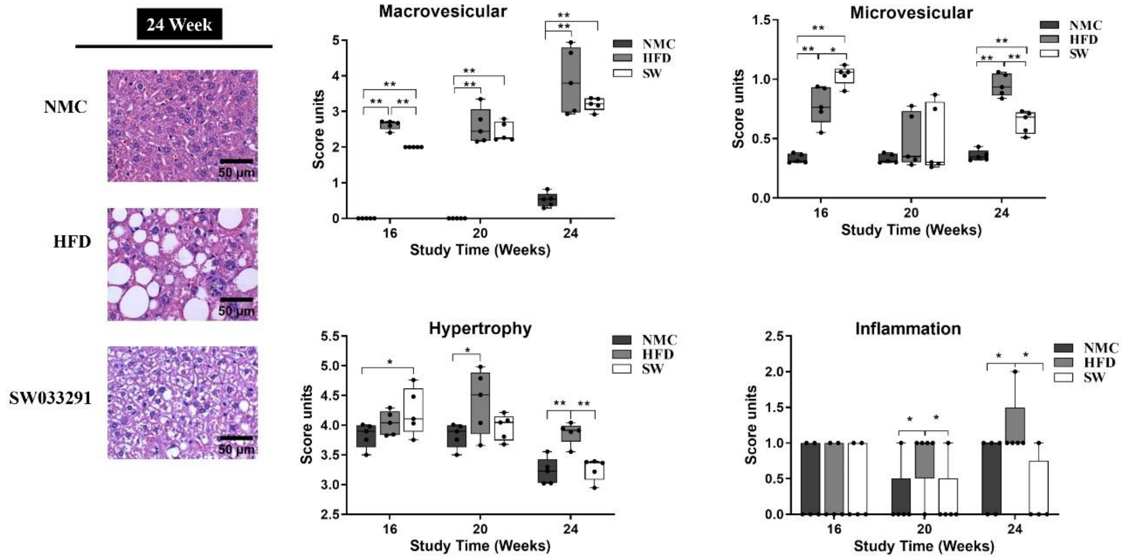

Figure S3b.

H&E –STAINING 16 WEEKS

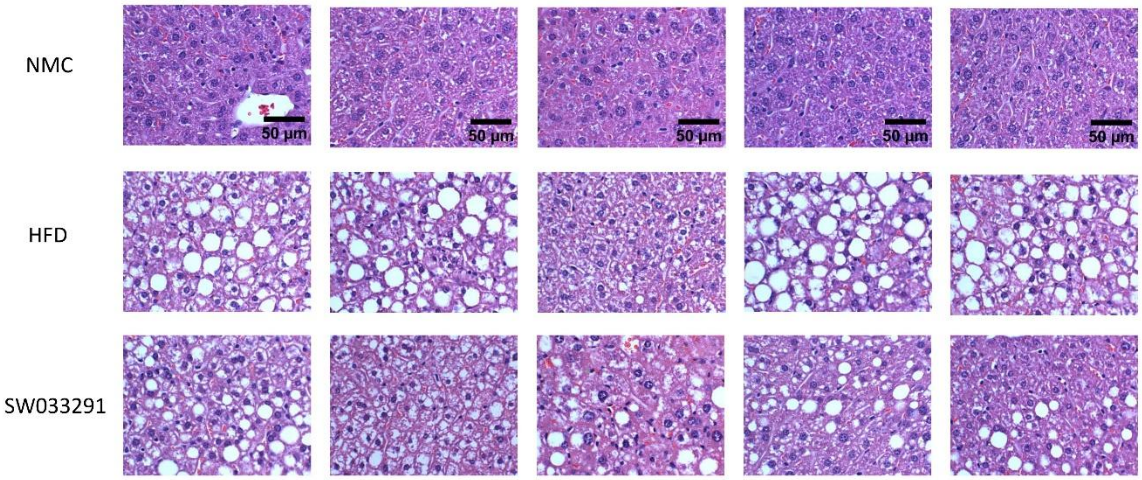

Figure S3c.

H&E –STAINING 20 WEEKS

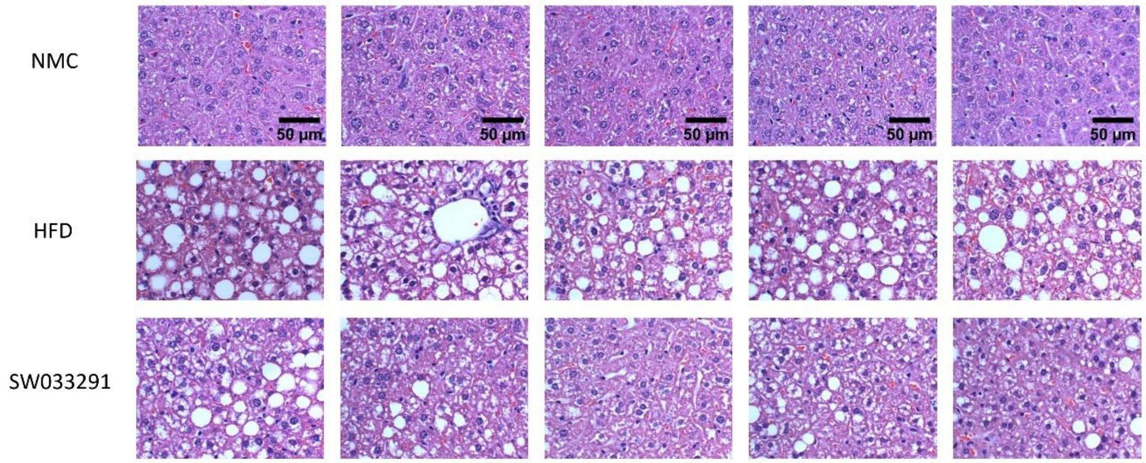

Figure S3d.

H&E –STAINING 24 WEEKS

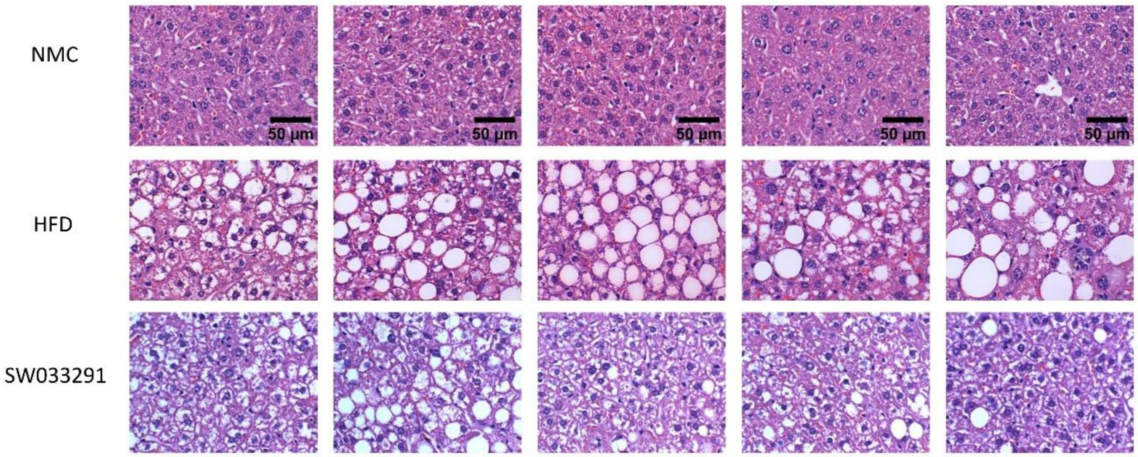

Figure S3. *Liver morphology assessment by the NAS score.* **a)** Representative images of H&E staining of liver tissues from each group were assessed for MASH development and progression. Results are as shown in the box and whisker charts (* $p < 0.05$, ** $p < 0.01$, by ANOVA and Tukey's post hoc test/t-test, $n = 5$). ***b)*** Representative H&E images of liver tissue from each mouse per experimental group at 16W ($n = 5$). ***c)*** Representative H&E images of liver tissue from each

mouse per experimental group at 20W (n = 5). ***d)*** Representative H&E images of liver tissue from each mouse per experimental group at 24W. (n = 5).

Figure S4a.

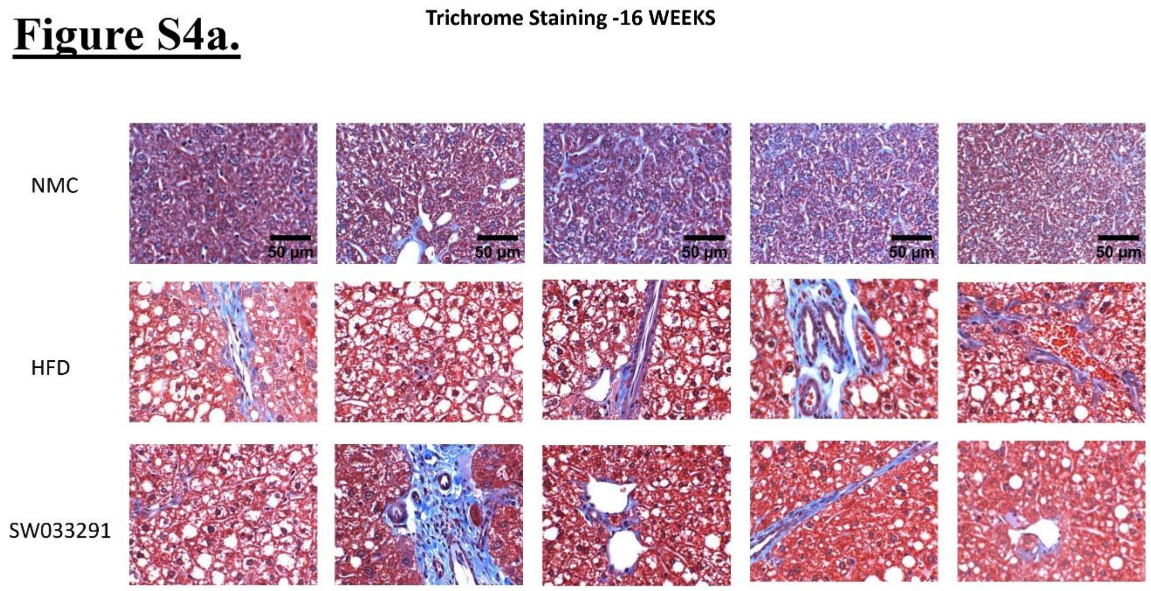

Figure S4b.

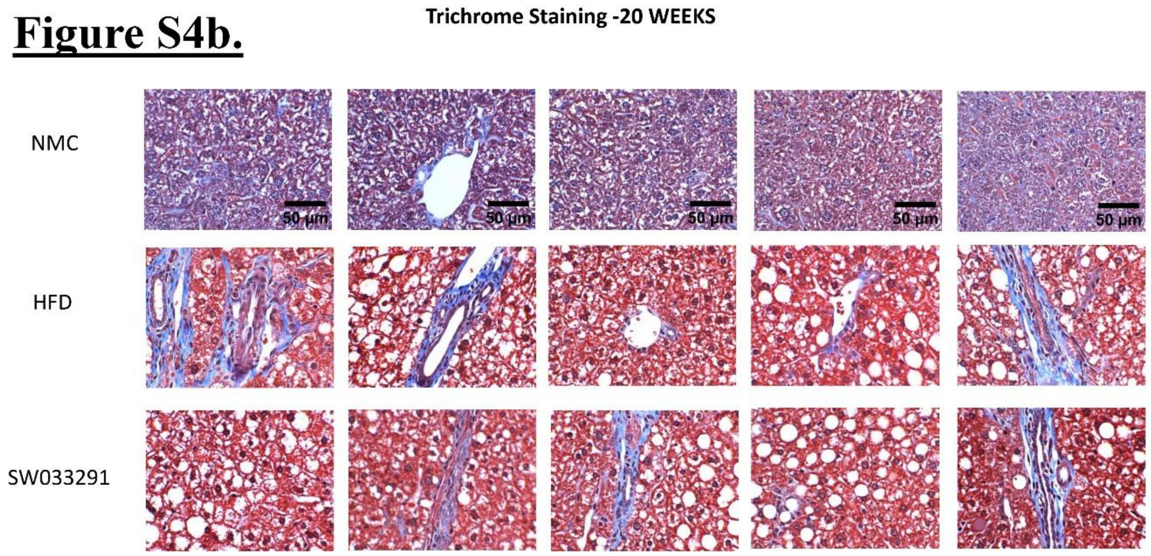

Figure S4c.

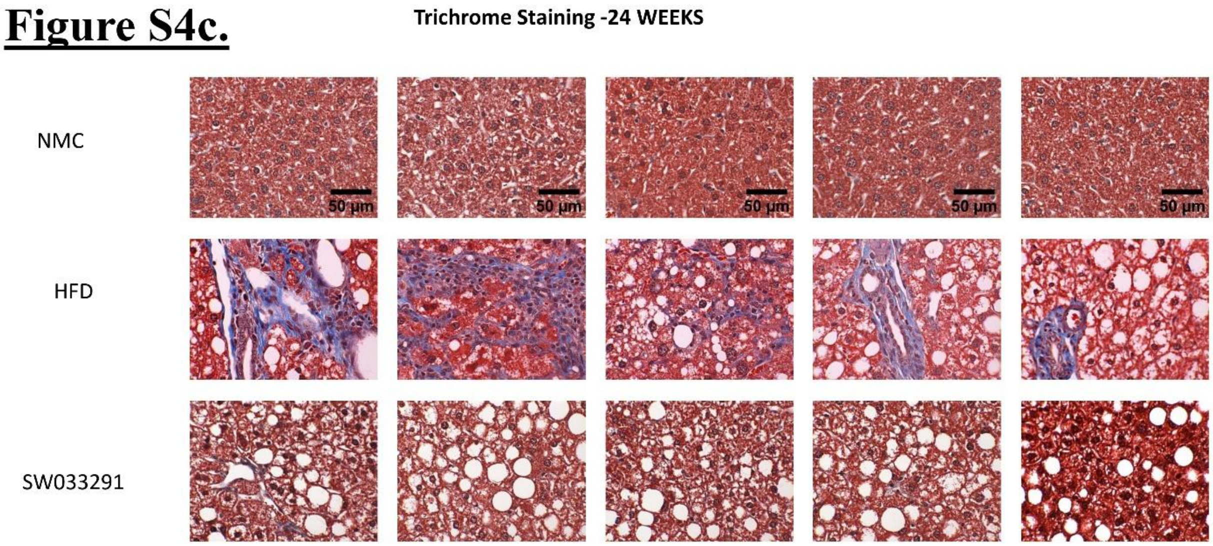

Figure S4. *Liver fibrosis assessment*. ***a)*** Representative trichrome images of liver tissue from each mouse per experimental group at 16W (n = 5). ***b)*** Representative trichrome images of liver tissue from each mouse per experimental group at 20W (n = 5). **c**) Representative trichrome images of liver tissue from each mouse per experimental group at 24W (n = 5).

Figure S5a.

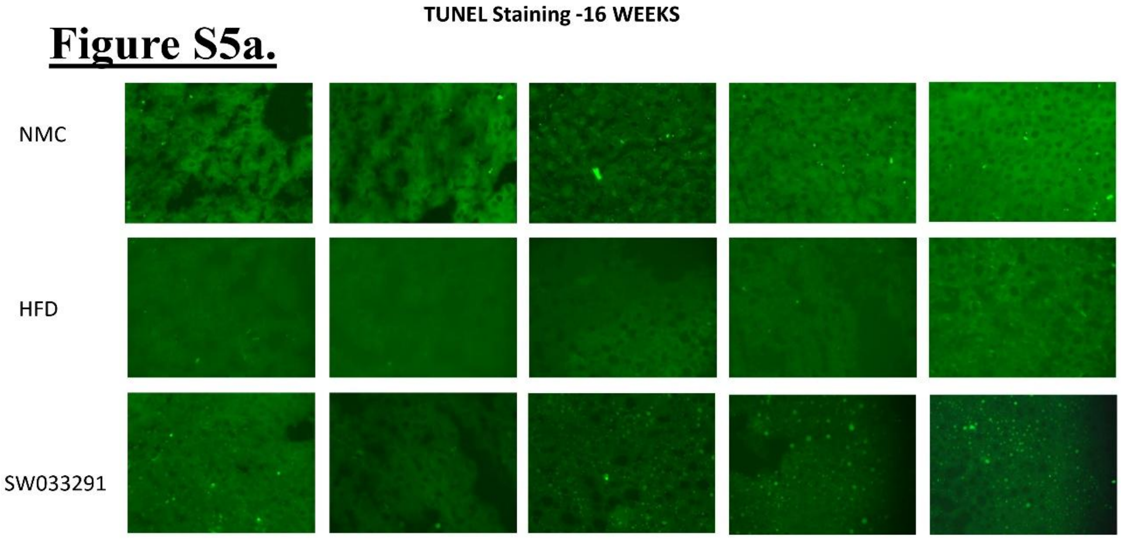

Figure S5b.

TUNEL Staining -20 WEEKS

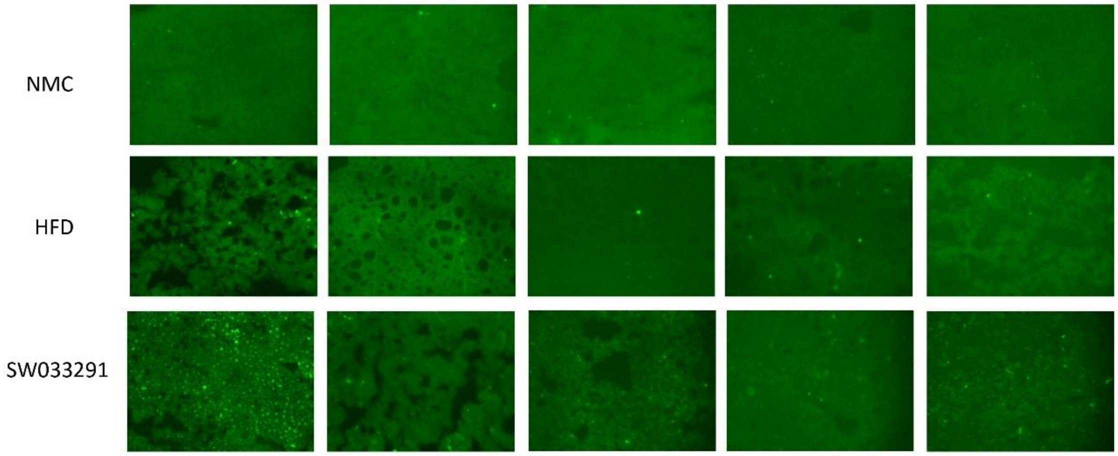

Figure S5c.

TUNEL Staining -24 WEEKS

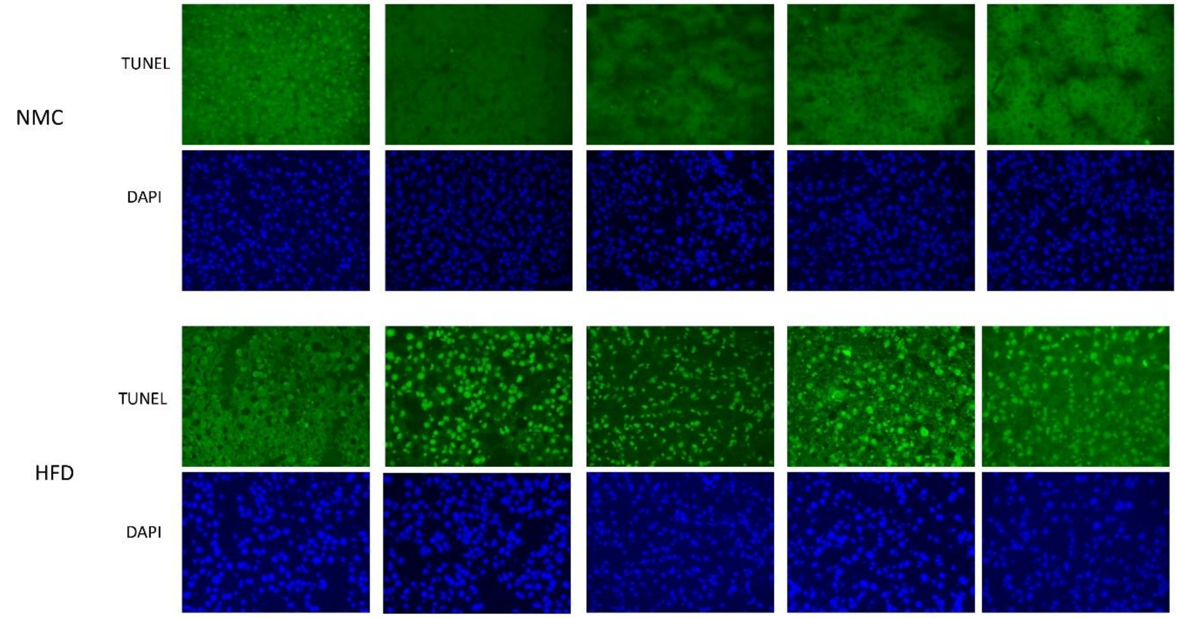

Figure S5c Contd.

TUNEL Staining -24 WEEKS CONTD

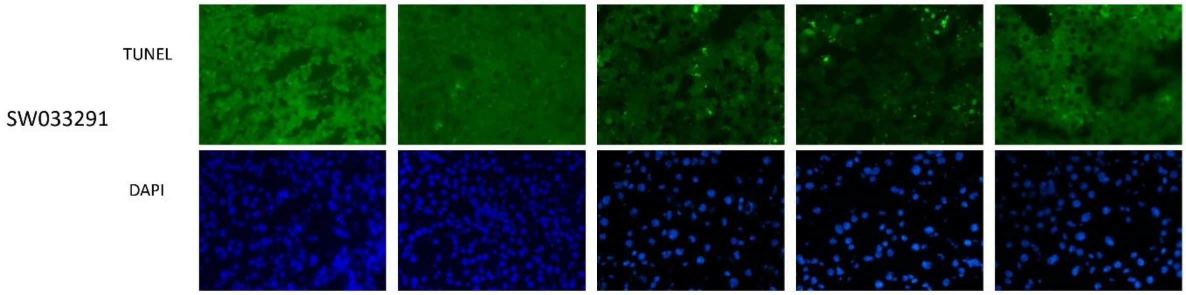

Figure S5. *Liver apoptotic activity assessment*. **a)** Representative TUNEL (apoptosis) images of liver tissue from each mouse per experimental group at 16W (n = 5). **b)** Representative TUNEL (apoptosis) images of liver tissue from each mouse per experimental group at 20W (n = 5). **c)** Representative TUNEL (apoptosis) and their corresponding nuclei staining (DAPI) images of liver tissue from each mouse per experimental group at 24W (n = 5).

Table S1.

Key reagents and chemical used in this study. Antibodies against the indicated protein as well as other reagents/chemicals used in this study are as listed below. Their catalogue number, source and the dilutions used in the experiments are as listed.

ANTIBODY/REAGENT/CHEMICAL	Source	Reference	Description	Host Sp.	IHC	WB
m-IgGκ BP-HRP	Santa Cruz Biotechnology	Catt sc-516102	Polyclonal Secondary	Mouse	N/A	1:5000
β-Actin (8H10D10) Mouse mAb3	Cell Signaling Technology	Catt 3700S	Monoclonal primary	Mouse	N/A	1:5000
FoxO1 (C29H4) Rabbit mAb	Cell Signaling Technology	Catt 2880S	Monoclonal primary	Rabbit	N/A	1:500
α-Tubulin Antibody	Cell Signaling Technology	Catt 2144S	Polyclonal primary	Rabbit	N/A	1:1000
Rabbit IgG HRP-conjugated Antibody	R&D systems	Catt HAF008	Polyclonal Secondary	Goat	N/A	1:1000
Click-iT Plus TUNEL Assay Kits for In Situ Apoptosis Detection	Thermofischer Scientific	Catt C10617	Detection kit	N/A	N/A	N/A
SirT7 (D3K5A) Rabbit mAb	Cell Signaling Technology	Catt 5360S	Monoclonal primary	Rabbit	N/A	1:1000
mTOR (7C10) Rabbit mAb	Cell Signaling Technology	Catt 2983S	Monoclonal primary	Rabbit	N/A	1:1000
